# Stress–strain characteristics of FRP–PVC confined spontaneous combustion gangue concrete columns

**DOI:** 10.1038/s41598-024-51928-5

**Published:** 2024-01-17

**Authors:** Jinyang Zhang, Haiqing Liu, Jinli Wang, Ming Lei, Zimu Chen

**Affiliations:** 1 School of Civil Engineering, Liaoning Technical University, Fuxin, 123000 China; 2 China Construction Fifth Engineering Division Corp., Ltd., Changsha, 410004 China

**Keywords:** Engineering, Civil engineering, Composites

## Abstract

A total of 40 fiber reinforced polymer (FRP) and polyvinyl chloride (PVC) confined spontaneous combustion gangue coarse-aggregate concrete (SAC) specimens were subjected to axial compression tests and theoretical studies. The main analysis focused on the impact of the replacement rate of spontaneous combustion gangue (SCG), the type of CFRP confinement, and the number of CFRP layers on the axial compression performance of CFRP–PVC confined SAC (CFRP–PVC–SAC). The results show that CFRP–PVC confinement can effectively enhance the axial compressive capacity, axial deformation, and lateral deformation of the components. The increase in strength ranges from 1.68 to 3.48 times, while the increase in strain ranges from 5.21 to 11.98 times. The crack patterns and expansive behavior of the coal gangue concrete under confinement exhibit significant differences compared to ordinary concrete. In addition, based on the framework of the existing FRP-confined plain concrete model, a modified model is established to facilitate prediction of stress–strain relationships for short columns of CFRP–PVC–SAC, with the calculated results in good agreement with experimental values.

## Introduction

In recent years, the mining of coal resources has produced a large amount of coal gangue and its long-term accumulation has caused land occupation, vegetation damage, water pollution and destruction, air pollution, heavy metal pollution, soil erosion, land desertification, and some other environmental problems^1–4^. To reduce the environmental pollution of coal gangue for rationalization, the current recycling technology of coal gangue is mainly brick burning, cement, concrete and paving, recovery of coal, pyrite, extraction of alumina, and preparation of anhydrous aluminum trichloride, crystalline aluminum chloride, polymeric aluminum chloride, and water glass^5–8^. Coal gangue is divided into spontaneous and nonspontaneous combustion gangue, with spontaneous combustion gangue involving the process of spontaneous combustion, with sulfide and nitride emissions through combustion. Its stability is far better than nonspontaneous combustion gangue^9,10^. Currently, global concrete use is huge, facing the problem of natural aggregate scarcity and, compared to traditional gangue utilization, the use of preparation in gangue concrete. Spontaneous combustion gangue used instead of stone as concrete coarse aggregate can reduce the use of natural sand and gravel can also reduce environmental pollution problems, in line with the concept of green and sustainable development^11–13^. Due to the high porosity of spontaneous combustion gangue, high water absorption, high pressure crushing value, and other defects, its inclusion in concrete strength is low, especially as durability is lower than natural aggregate concrete^14–17^. To solve the performance problems of gangue concrete, the use of external constraints to improve the original compressive properties of concrete has changed from one-way pressure to three-way pressure, which can improve gangue concrete bearing capacity as well as ductility.

Fiber reinforced polymer (FRP), a new environment-friendly fiber material developed in recent years, has the advantages of good durability, lightweight, high strength, and easy construction, and is increasingly used in civil engineering^18–22^. Zhao et al.^23^ have studied the replacement rate of FRP-constrained recycled aggregate concrete and found that, when the replacement proportion of recycled aggregate is 20%, the mechanical properties of specimens were similar to those of ordinary concrete, while the strength of specimens at 100% recycled aggregate replacement was lower. Zhou et al.^24^ have studied carbon-FRP (CFRP)-constrained light-aggregate concrete and found significantly improved load-bearing capacity and ductility. Also, CFRP-constrained concrete significantly improved the interfacial properties of steel balls with the cement matrix compared to unconfined concrete, maximizing the mechanical properties of light-aggregate concrete. Zeng et al.^25^ have studied CFRP-constrained recycled glass aggregate concrete, recycled glass aggregates replaced coarse and fine aggregates separately. The fine aggregate replacement exhibited little effect, coarse aggregate replacement affected strength, and the number of CFRP layers positively correlated with the strength and ductility of the specimens. The stress–strain characteristics of FRP-constrained concrete with different types of aggregates are significantly different compared to FRP-constrained plain concrete.

Due to its corrosion resistance, polyvinyl chloride (PVC) is used as a permanent formwork for constrained concrete^26–28^. PVC is a commonly used construction material, having huge production, low price, excellent mechanical properties, certain flame retardant ability, and is generally used to make various profiles, pipes, and plates for civil and industrial use, with strong corrosion resistance^29–33^. Studies have shown that PVC materials maintain better performance under harsh environments, such as salts, chloride ions, freezing, and thawing^34–37^. Combining FRP and PVC could bring the advantages of both confining materials into play. If there is no PVC pipe and the FRP strip is wound directly on the concrete column, the FRP strip will not confine the concrete uniformly, leading to early damage to the concrete between strips during the loading process^39^. Gao et al.^38^ have investigated FRP–PVC-constrained RAC concrete and the composite enclosure system found to significantly improve compressive strength and axial and lateral deformation of RAC–RCBA, thus establishing a design-oriented compressive stress–strain model for PFRP–PVC–RAC–RCBA specimens. Fang et al.^39^ have conducted PVC–CFRP pipe-reinforced concrete short column axial compression tests and derived calculated equations for the bearing capacity of PVC–CFRP pipe concrete columns according to the static equilibrium conditions and ultimate equilibrium conditions. For the FRP–PVC constrained form, the inner PVC material can be used as the permanent formwork of gangue concrete members and it can also make the gangue concrete free from the external bad environment, play a role of isolation and protection for the core concrete, and give full play to the confining effects of the external FRP. The outer FRP can greatly improve the axial compression limit strain of core gangue concrete and can improve the bearing capacity of gangue concrete to a certain extent.

This paper analyzes the force process of the specimens, discusses their failure modes, and elucidates the differential compressive behavior and stress–strain relationships of SAC and NAC under the CFRP–PVC composite confinement. Using existing theoretical models of axial compression ultimate strength and ultimate strain of constrained concrete for comparison and calculation, using data fitting methods to establish a more accurate model, which is then applied to CFRP–PVC–SAC.

## Experimental design

### Specimen design

**Figure 1:**
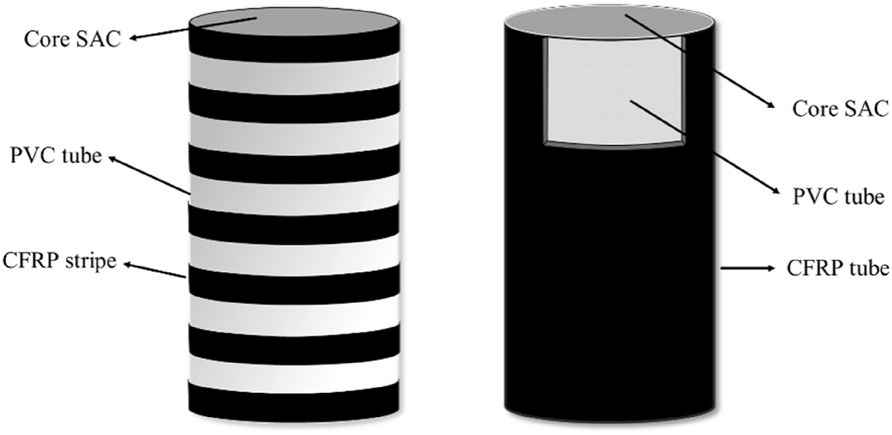
CFRP–PVC–SAC schematic diagram.

A total of 40 specimens were designed for this test, including 8 unconfined cylindrical specimens, 16 CFRP–PVC strip spacing confinement with a strip width of 25 mm and spacing of 20.8 mm, and 16 CFRP–PVC full-wrap confinement (Fig. 1). The cylindrical specimens were all short columns, 150 mm in diameter and 300 mm in height (Table 1). Each concrete column was cured for 28 days and two identical specimens were designed for each type to ensure repeatability of test results. To prevent early damage at the ends of the constrained specimens, 2 additional layers of CFRP strips with a width of 25 mm were pasted on the ends of all constrained specimens. In the specimen name, C is CFRP, P means PVC, 3 represents the number of CFRP layers as 3 layers, S20 a spacing of 20.8 mm, R the coarse aggregate SCG replacement proportion. Thus, C3PS20R30-1 indicated the CFRP–PVC strip spacing of 20.8 mm constraint, spontaneous combustion gangue replacement of 30%, and the first of two identical specimens.

**Table 1:** Design parameters of specimens.

Specimen	*D* (mm)	*H* (mm)	*r* (%)	*t* (mm)	CFRP constraint form
C1PS20R0	150	300	0	0.167	Spacing distance 20.8 mm
C3PS20R0	150	300	0	0.501	Spacing distance 20.8 mm
C1PR0	150	300	0	0.167	Tube
C3PR0	150	300	0	0.501	Tube
C1PS20R30	150	300	30	0.167	Spacing distance 20.8 mm
C3PS20R30	150	300	30	0.501	Spacing distance 20.8 mm
C1PR30	150	300	30	0.167	Tube
C3PR30	150	300	30	0.501	Tube
C1PS20R60	150	300	60	0.167	Spacing distance 20.8 mm
C3PS20R60	150	300	60	0.501	Spacing distance 20.8 mm
C1PR60	150	300	60	0.167	Tube
C3PR60	150	300	60	0.501	Tube
C1PS20R100	150	300	100	0.167	Spacing distance 20.8 mm
C3PS20R100	150	300	100	0.501	Spacing distance 20.8 mm
C1PR100	150	300	100	0.167	Tube
C3PR100	150	300	100	0.501	Tube

### Mechanical properties of materials

**Figure 2:**
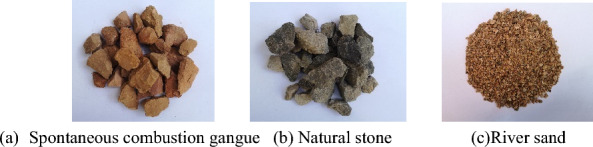
Appearance of coarse and fine aggregates.

The coarse aggregate of spontaneous combustion gangue (SCG) was obtained from crushing, washing, and grading SCG in a mining area of Liaoning, China, and natural coarse aggregate common crushed stone. PO42.5 grade ordinary silicate cement, mixing water for city tap water, fine aggregate for ordinary river sand. The coarse and fine aggregates used in the test are shown in Fig. 2. According to (JGJ52-2006)^40^, the physical and mechanical properties of spontaneous combustion coal gangue aggregates and natural aggregates are shown in Table 2. Due to the high water absorption of SCG coarse aggregate, in coarse-aggregate concrete (SAC) preparation, additional water was added to SCG coarse aggregate as advance wetting. In accordance with (JGJ55-2011)^41^, the specific concrete mixes are shown in Table 3. According to the ASTM standard^42^ and (ASTM D3039-2008) tensile tests on FRP flat sheet, the tensile mechanical properties of FRP and PVC were derived and the specific parameters shown in Table 4.

**Table 2:** Physical and mechanical properties of spontaneous combustion gangue and natural aggregate.

Material characteristics	Spontaneous combustion gangue	Natural stone aggregate
Apparent density (kg/m^3^)	2427	2657
Water absorption (%)	9.6	2.3
Moisture content (%)	0.82	1.1
Crushing index (%)	20.68	5.94
Tube crushing strength (MPa)	5.4	32.5

**Table 3:** Concrete mix ratio.

No.	Replacement (%)	*w/c*	Cement (kg/m^3^)	Water (kg/m^3^)	Sand (kg/m^3^)	Natural stone aggregate (kg/m^3^)	Spontaneous combustion gangue (kg/m^3^)	Additional water (kg/m^3^)	Superplasticizer (kg/m^3^)
1	0	0.43	363	156	959	922	0	0	6.53
2	30	0.43	363	156	959	645	277	21.3	6.53
3	60	0.43	363	156	959	369	553	41	6.53
4	100	0.43	363	156	959	0	922	88.74	6.53

**Table 4:** Mechanical properties of materials.

Confining materials	Thickness (mm)	Elastic modulus (GPa)	Tensile strength (MPa)
CFRP	0.167	245.0	3870.0
PVC	5	2.7	43.2

### Specimen preparation

All unconfined specimens were cast in plastic molds of 150 mm diameter and 300 mm height for concrete specimens. For constrained specimens, concrete was directly filled into the PVC pipe, and the top and bottom surfaces of all specimens polished smooth and flat to ensure uniform loading. The top end was sealed with plastic film and all specimens maintained under the same conditions for 28 days. Before pasting on CFRP strip, specimen surfaces were wiped with water and the CFRP strip pasted on the specimens with a matching epoxy resin adhesive. Two layers of CFRP strip with 25 mm width and 0.167 mm thickness were applied at both ends to avoid premature rupture due to stress concentration. The length of CFRP in the overlapping area was 150 mm.

### Test Equipment and test methods

**Figure 3:**
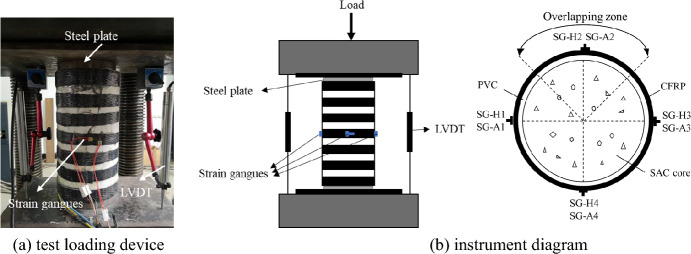
Test loading device and instrument diagram.

Eight strain gauges were arranged in the middle of the specimen to measure specimen longitudinal and circumferential strains. One displacement gauge was also arranged on each side of the specimen to measure specimen axial displacement (Fig. 3). The test was performed using a 5000 kN hydraulic testing machine for axial compression of all specimens. The loading method was partly section loading. All test data were recorded by a Dynamic Signal Acquisition and Analysis System (DHDAS; Dong-hua Testing Technology Co., Ltd., Taizhou, China), including load, displacement, and strain. In each test, the specimens were first preloaded to ensure that the strain gages and linear variable displacement transducers (LVDTs) were properly connected and calibrated and then concentric loading determined by observing axial strain gauge readings during the preloading phase.

## Test results and discussion

### Unconstrained specimens

The axial compression damage of the unconfined specimens showed that plain concrete usually exhibited a large number of microcracks accompanied by spalling of the concrete blocks (Fig. 6). In contrast, the SAC exhibited brittle damage, with wide vertical cracks on each cylindrical surface.

**Figure 4:**
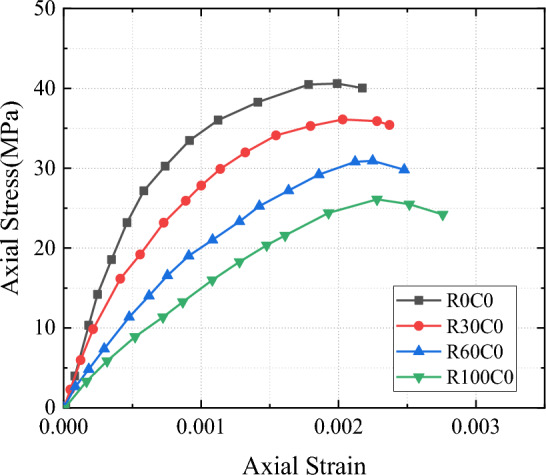
Axial stress–strain curves of unconstrained cylindrical specimen.

The stress–strain curves of unconfined SAC were compared with those of ordinary concrete (Fig. 4). SAC is very different from ordinary concrete in many aspects. First, the slope of the stress–strain curve in the initial stage was smaller than that of ordinary concrete, which was because of the apparent density of SCG (i.e., 2427 kg/m^3^) was lower than that of natural aggregate (i.e., 2657 kg/m^3^). Because the elastic modulus of concrete is positively correlated with the apparent density of aggregate, the low apparent density of SCG leads to the low elastic modulus of SAC. Second, the peak strain of SAC was greater than that of natural aggregate concrete (NAC), which indicated that the deformation capacity of SAC was greater than that of NAC. This phenomenon also indicated that brittle damage occurred in SAC and the compressive strength of specimens with a 30% replacement was 11.1% lower than that of normal concrete specimens. Also, the strength of specimens with a 100% replacement proportion was 35.7% lower than that of normal concrete specimens. This indicated that the incorporation of a small amount of SCG did not have a significant weakening effect on concrete strength, while too high a content of SCG significantly reduced concrete strength.

### Constrained specimens

#### Failure mode

**Figure 5:**
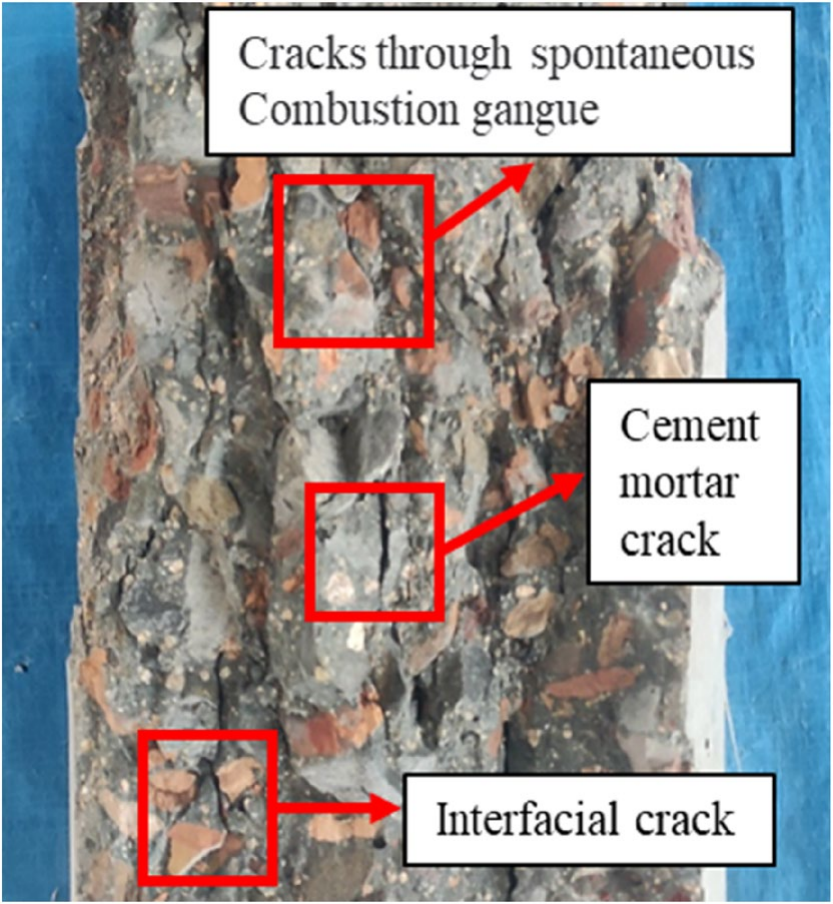
Internal failure patterns of CFRP–PVC-bound samples.

**Figure 6:**
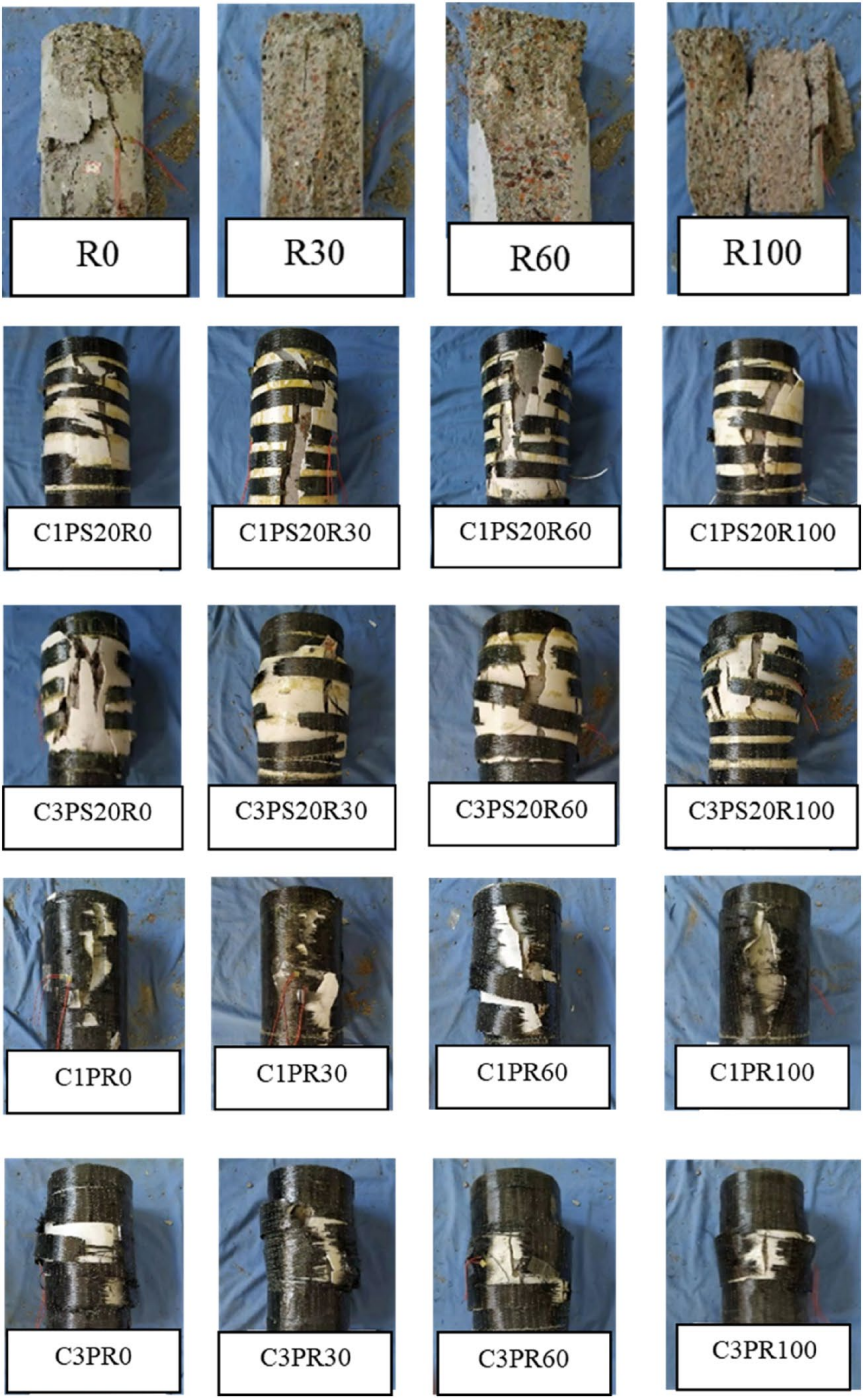
Failure morphology of specimens.

CFRP–PVC fully wrapped 1-layer specimens showed CFRP tearing and PVC damage with long penetration cracks, as in the case of specimen C1PR100 (Fig. 6). The damage of the CFRP fully-wrapped 3-layer specimens was concentrated in the middle of the specimen, due to the clear lateral expansion of internal SAC causing the PVC pipe to bulge significantly, as in the case of specimen C3PR60. The stronger external confinement provided by the 3-layer CFRP only caused the middle CFRP to have PVC damage. The change in SCG replacement proportion did not have a significant effect on the specimen failure mode. The anatomy of the concrete inside the constrained specimen showed the interface damage, cement mortar cracks, and interface cracks, which are common in normal concrete (Fig. 5). However, in addition to these cracks, there were also cracks through the SCG. This was because SCG was a porous material with much higher porosity than natural aggregates and had a cylinder compressive strength of 5.42 MPa for SCG, which was much lower than that of natural stone. Once the aggregate was crushed, the internal structure of the concrete was destroyed.

The damage pattern of the CFRP strip-constrained specimens was different from that of the CFRP fully wrapped-constrained specimens (Fig. 6). CFRP strip-constrained specimens had circumferential cracks and multiple longitudinal cracks at the PVC pipe, while longitudinal cracks did not penetrate the entire height of the PVC pipe, as in the case of specimen C1PS20R100. The hoop-like rupture of the PVC pipe was attributed to the local strengthening effect of the CFRP strips on the specimens. It was further observed that, for the constrained specimens in the form of CFRP strips, increased CFRP strip thickness did not change specimen damage patterns.

Compared with unconfined concrete specimens, the strength of the CFRP–PVC-confined specimens was increased by 1.68–3.4 times (Table 5). The ultimate strain of each specimen increased by 5.21–11.98 times and the enhancement effect increased with increased SCG replacement proportion. Compared with unconfined specimens, the axial compressive strength and axial ultimate strain were both improved, with the axial ultimate strain of the specimens especially improved.

**Table 5:** Test parameter results.

Specimen	$\varepsilon_{co}$(%)	$f_{co}$(MPa)	$\varepsilon_{h,rup}$(%)	Avg	$f_{l}$(MPa)	$f_{cu}$(MPa)	Avg	${{f_{cu} } \mathord{\left/ {\vphantom {{f_{cu} } {f_{co} }}} \right. \kern-0pt} {f_{co} }}$	$\varepsilon_{cu}$(%)	Avg	${{\varepsilon_{cu} } \mathord{\left/ {\vphantom {{\varepsilon_{cu} } {\varepsilon_{co} }}} \right. \kern-0pt} {\varepsilon_{co} }}$
C1PS20R0-1	0.199	40.6	1.05	1.02	5.88	71.4	68.1	1.68	1.06	1.04	5.21
C1PS20R0-2	0.199	40.6	0.99			64.8			1.01		
C3PS20R0-1	0.199	40.6	1.21	1.16	13.13	93.5	90.9	2.24	1.66	1.60	8.04
C3PS20R0-2	0.199	40.6	1.11			88.4			1.54		
C1PR0-1	0.199	40.6	1.13	1.07	8.36	78.6	76.2	1.88	1.29	1.20	6.05
C1PR0-2	0.199	40.6	1.01			73.8			1.12		
C3PR0-1	0.199	40.6	1.33	1.29	22.71	118.5	117.3	2.89	2.14	2.17	10.89
C3PR0-2	0.199	40.6	1.25			116.1			2.19		
C1PS20R30-1	0.203	36.1	1.07	1.00	5.81	65.9	63.8	1.77	1.18	1.10	5.40
C1PS20R30-2	0.203	36.1	0.93			61.7			1.01		
C3PS20R30-1	0.203	36.1	1.19	1.13	12.86	90.6	87.9	2.43	1.74	1.68	8.25
C3PS20R30-2	0.203	36.1	1.07			85.1			1.61		
C1PR30-1	0.203	36.1	1.16	1.09	8.46	72.4	70.5	1.95	1.37	1.29	6.35
C1PR30-2	0.203	36.1	1.02			68.6			1.21		
C3PR30-1	0.203	36.1	1.24	1.19	21.16	109.5	108.3	3.00	2.36	2.33	11.47
C3PR30-2	0.203	36.1	1.14			107.1			2.30		
C1PS20R60-1	0.212	30.8	1.01	0.97	5.73	57.7	54.4	1.77	1.19	1.11	5.22
C1PS20R60-2	0.212	30.8	0.93			51.1			1.02		
C3PS20R60-1	0.212	30.8	1.11	1.08	12.38	84.5	81.1	2.63	1.91	1.84	8.66
C3PS20R60-2	0.212	30.8	1.05			77.6			1.76		
C1PR60-1	0.212	30.8	1.07	1.02	8.11	67.5	64.8	2.10	1.46	1.39	6.56
C1PR60-2	0.212	30.8	0.97			62.1			1.32		
C3PR60-1	0.212	30.8	1.16	1.11	19.91	102.9	100.5	3.26	2.56	2.46	11.60
C3PR60-2	0.212	30.8	1.06			98.1			2.36		
C1PS20R100-1	0.228	26.1	1.01	0.95	5.68	51.4	50.3	1.93	1.34	1.28	5.61
C1PS20R100-2	0.228	26.1	0.89			49.2			1.22		
C3PS20R100-1	0.228	26.1	1.09	1.07	12.27	74.5	71.8	2.75	2.08	2.02	8.84
C3PS20R100-2	0.228	26.1	1.05			69.1			1.95		
C1PR100-1	0.228	26.1	1.03	1.01	8.06	58.9	57.3	2.20	1.61	1.56	6.86
C1PR100-2	0.228	26.1	0.99			55.7			1.52		
C3PR100-1	0.228	26.1	1.15	1.10	19.68	93.5	90.7	3.48	2.81	2.73	11.98
C3PR100-2	0.228	26.1	1.05			87.9			2.65		

#### SCG replacement rate impact

**Figure 7:**
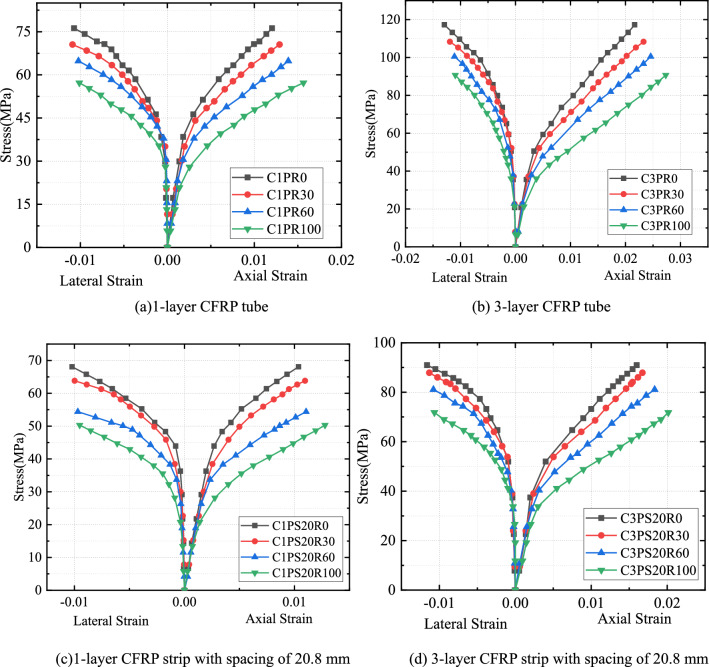
Effects of replacement rate on stress–strain properties of CFRP–PVC confined concrete.

**Figure 8:**
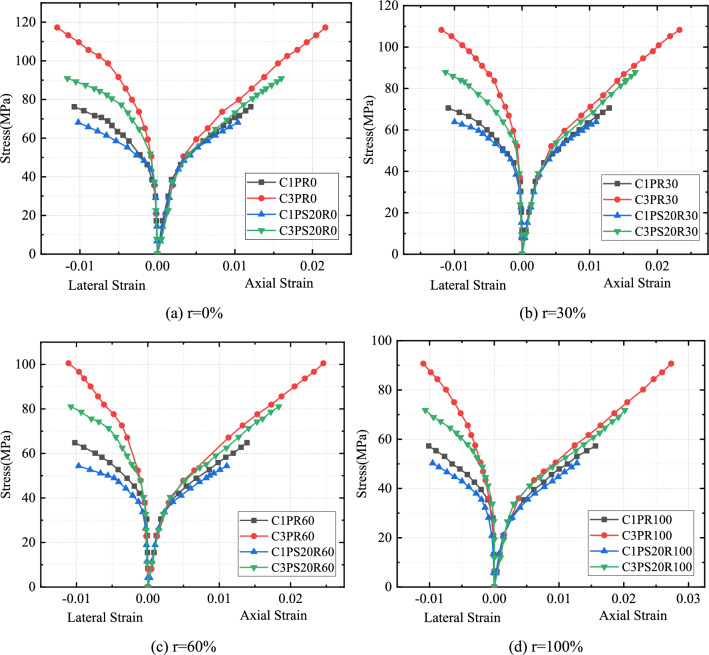
Effects of external constraints on stress–strain properties of CFRP–PVC-constrained concrete.

Axial and circumferential stress–strain relationships for all CFRP-confined specimens showed that the stress–strain curves of all specimens almost showed a bilinear characteristic (Figs. 7 and 8). These curves include approximately linear rising segments, coupled with a transition zone and another linear rising part.

The effects of the SCG replacement proportion on the stress–strain curve for the same CFRP thickness showed that, with increased replacement ratio, the initial strength of concrete gradually decreased and the transition zone of CFRP-confined SAC smoother and flatter in the second stage (Fig. 7). Meanwhile, increased replacement led to decreased ultimate compressive strength and increased ultimate compressive strain in specimens. Compared with plain concrete specimens under the same confinement, the compressive strength was reduced by 3.3–26.1% and the ultimate axial strain increased by 4.7–29.9%. The porous nature, low density, and friability of SCG, as well as its compressive strength (5.4 MPa), was much lower than that of natural aggregates (30–40 MPa), resulting in a decrease in the initial stiffness of the stress–strain curve. As a result of the SCG admixture, the axial compression produced larger voids inside the concrete, thus increasing the axial compression deformation of the specimens.

#### CFRP spacing influence

The stress–strain curves of CFRP strip PVC–SAC showed a similar overall trend to that of CFRP full-wrap-PVC–SAC (Fig. 8). At the same CFRP thickness, the initial rising stage of the CFRP strip-constrained specimens was the same as the initial rising stage of the CFRP fully-wrapped specimens and the slope of the second rise phase of the CFRP strip-confined compared to the CFRP full-wrap-confined became lower due to the reduced confinement stiffness. Due to the confinement form of CFRP strips, spacing reduced the local confinement effect of the specimen, with the ultimate stress and ultimate strain of the CFRP strip confinement specimen reduced by the influence of CFRP strip confinement.

#### CFRP thickness effect

The strength and ultimate strain of both SAC and NAC were significantly increased under CFRP confinement and the ultimate strength and ultimate strain of the confined specimens positively correlated with CFRP thickness (Fig. 8). The first-stage curves of specimens with different CFRP tube thicknesses did not differ significantly. In the initial stage, the force characteristics were the same and the core concrete of each specimen less affected by circumferential confinement. The confinement effect on the core concrete was very small and CFRP had little effect on specimens at the early stage of loading due to the slow development of cracks inside the concrete and small transverse deformation. After entering the transition section, microcracks inside the concrete developed continuously and the strain growth rate of the specimen accelerated. The stress strain at this stage was mainly influenced by CFRP thickness. With the increase in the thickness of the CFRP tube, the stress–strain curve gradually smoothed in the transition zone, and the slope of the second stage increased, along with an increase in axial strain. The larger the CFRP thickness, i.e., the greater the CFRP confinement stiffness, the slower the CFRP strain growth rate. Afterward, the curve approximated a straight line, and deformation tended to become stable. In summary, the results of the second part of the stress–strain curve depended largely on the confining stiffness and concrete type.

## Dilation properties

**Figure 9:**
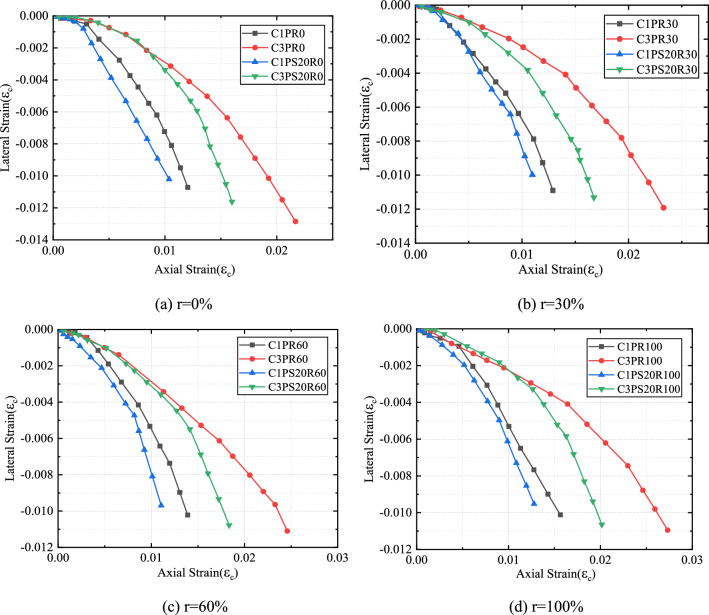
Effects of external constraints on axial strain-lateral strain curves of CFRP–PVC-constrained concrete.

**Figure 10:**
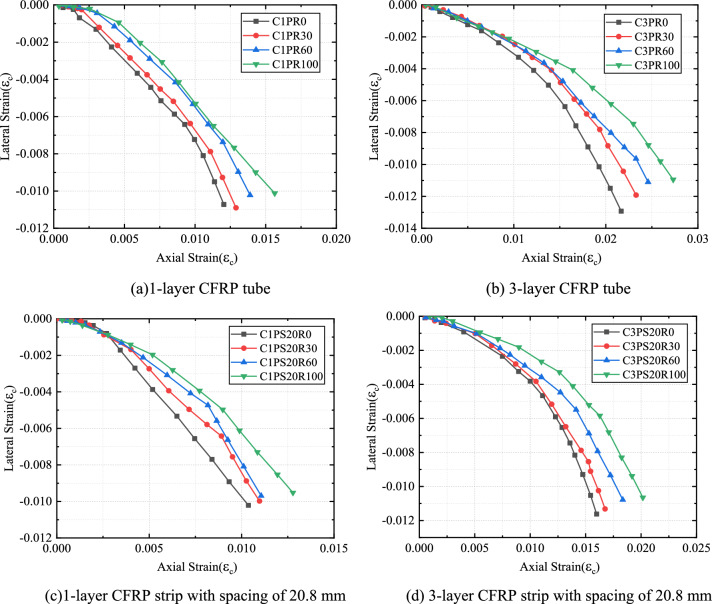
Effects of replacement rate on axial strain-lateral strain curves of CFRP–PVC-confined concrete.

The expansion characteristics of CFRP-confined concrete were reflected by the axial strain-lateral strain relationship. The lateral-axial strain relationships of all specimens are shown in Figs. 9 and 10. The overall trend of the curves showed a bilinear pattern, in which the expansion rates of specimens were small in the initial stage of deformation and started to increase rapidly when the axial strain approached the strain corresponding to the peak stress of unconfined concrete. Next, the magnitude of the lateral strain increased almost linearly with increased axial strain. The change in external confinement was seen to have a greater effect on the swelling characteristics of the specimens compared to the change in internal aggregate.

Under the same SCG replacement proportion, the larger the CFRP stiffness was, the smaller the expansion rate and the smoother the axial strain-lateral strain curve under a certain axial strain (Fig. 9). Strip confinement and full-wrapping confinement cases were compared and the full-wrapped confinement form had a smaller expansion rate under the same conditions.

SAC underwent expansion after a certain delay, leading to a subsequent delay in activating the FRP confinement effect and the delay more significant as SCG content increased (Fig. 10). For the same CFRP thickness, the transverse strain of SAC was relatively lower than that of NAC for the same axial strain. This phenomenon was explained as due to: (1) brittle damage of SCG changed the nature of cracks appearing in the concrete, from microcracks to large local cracks; and (2) porosity, low density, and friability of SCG led to delayed dilation under FRP confinement. The damage of porous aggregate indicated that core concrete contained a high degree of internal space, with its volume expansion thus suppressed and lateral expansion reduced under FRP confinement. The lateral expansion of SAC was lower than that of NAC and the confinement effect of CFRP on SAC was significant.

## Constraint model

### Analysis of CFRP–PVC–SAC mechanical properties under pressure

**Figure 11:**
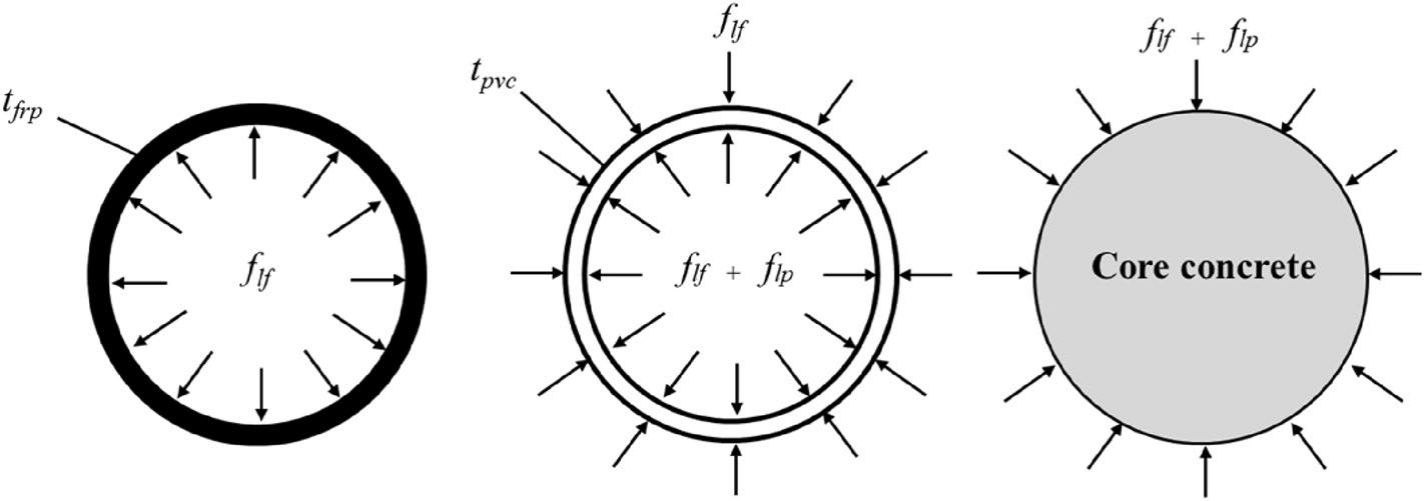
Mechanical properties of CFRP–PVC-confined concrete cylinders.

To design CFRP–PVC–SAC structures suitable for practical applications, an accurate stress–strain model was required. To establish the stress–strain model, it was important to understand the mechanical properties of CFRP–PVC–SAC under axial compression. In this section, based on the mechanical analysis of CFRP–PVC-confined concrete introduced by Yu et al., the mechanical properties of CFRP–PVC–SAC under axial pressure were analyzed^43^. By similar analysis, the axial compressive properties of CFRP–PVC–SAC were evaluated (Fig. 11). The core concrete was in triaxial compression, which enhanced the load-bearing capacity and deformation capacity of the concrete. Both CFRP and PVC provided lateral surround pressure for the core concrete, as shown in Eq. (1), expressed as

1 $$f_{l} = f_{lf} + f_{lp} .$$

The force balance in the PVC pipe was expressed in Eq. (2) and the force balance in the CFRP section by Eq. (3)^44^ as (Fig. 11)

2 $$f_{lp} = \frac{{2E_{PVC} \varepsilon_{PVC} t_{PVC} }}{d}\;{\text{and}}$$

3 $$f_{lf} = \frac{{2E_{frp} \varepsilon_{hfrp} t_{frp} }}{{d + t_{PVC} }},$$

where $f_{l}$ is the lateral circumferential pressure of the composite-constrained core concrete, $f_{lf}$ the effective lateral circumferential pressure provided by CFRP, $f_{lp}$ the lateral circumferential pressure provided by PVC, $E_{PVC}$, $\varepsilon_{PVC}$, and $t_{PVC}$ the modulus of elasticity, circumferential strain, and thickness of PVC pipe, respectively, $E_{frp}$, $\varepsilon_{hfrp}$, and $t_{frp}$ the modulus of elasticity, circumferential strain at rupture, and thickness of CFRP, respectively, and *d* the diameter of the core concrete.

For a CFRP strip–PVC constrained cylinder, the CFRP equivalent thickness $t^{\prime}_{frp}$ was expressed by Eq. (4) as

4 $$t^{\prime}_{frp} = \frac{{nw_{frp} }}{H}t_{frp} .$$

**Figure 12:**
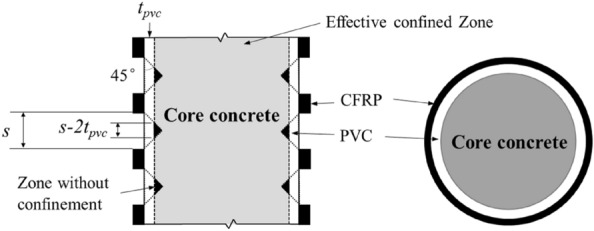
Mechanical properties of concrete columns confined by CFRP strip-PVC.

The force balance of the CFRP strip confinement was expressed in Eq. (5) as

5 $$f_{lf} = \frac{{2nE_{frp} \varepsilon_{hfrp} w_{frp} t_{frp} }}{{H\left( {d + t_{PVC} } \right)}},$$

where *n* is the number of CFRP strips, $w_{frp}$ the width of CFRP strips, and *H* is the height of a concrete column. The lateral circumferential pressure provided by CFRP strips was applied to the concrete core through the PVC with a dispersion angle of 45° and a dispersion width of the thickness of the PVC pipe (Fig. 12). Thus, the effective lateral envelope pressure of the concrete core was expressed as

6 $$f_{l,a} = k_{e} f_{l} ,$$

7 $$k_{e} = \frac{{A_{e} }}{A},$$

8 $$A_{e} = A - m\frac{{\left( {s - 2t_{pvc} } \right)^{2} }}{2},\;{\text{and}}$$

9 $$A = dH.$$

where $k_{e}$ is the effective confinement coefficient of CFRP strips, $k_{e} = 1$ the CFRP pipe all-inclusive confinement, $A_{e}$ the effective constrained area of core concrete, *A* the total area of the specimen including core concrete and external confinement, *m* the number of unconstrained areas between CFRP strips and *s* the spacing of CFRP strips. In summary, Eq. (1) can be expressed in Eq. (10) as

10 $$f_{l,a} = f_{lf} + f_{lp} = k_{e} f_{lf} + f_{lp} .$$

Specifically, for CFRP (fully wrapped) pipes–PVC-constrained columns, Eq. (10) became Eq. (11), expressed as

11 $$f_{l,a} = \frac{{2E_{frp} \varepsilon_{frp} t_{frp} }}{{d + 2t_{PVC} }} + \frac{{2E_{PVC} \varepsilon_{PVC} t_{PVC} }}{d}.$$

For CFRP strips-PVC-bound cylinder, Eq. (12) was developed as

12 $$f_{l,a} = k_{e} \frac{{2nE_{frp} \varepsilon_{frp} w_{frp} t_{frp} }}{{H\left( {d + 2t_{PVC} } \right)}} + \frac{{2E_{PVC} \varepsilon_{PVC} t_{PVC} }}{d}.$$

The *k*_*g*_ is the coefficient of the enclosing pressure effect of CFRP tape^39^, defined as:

13 $$k_{g} = \frac{{s_{f} }}{s}$$

where *s*_*f*_ denotes the width of the CFRP strip, *s* the spacing of the CFRP strip, and when $s \ge s_{f}$,$k_{e} = k_{g} \frac{{A_{e} }}{A}.$

### Stress–strain model correction

**Figure 13:**
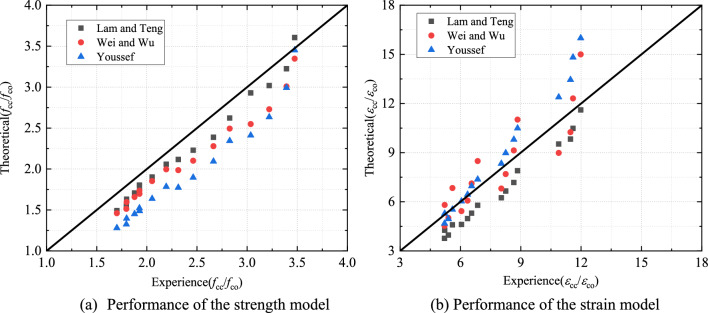
Performance of existing models.

In this section, three models were selected to predict the ultimate stress and strain of CFRP–PVC–SAC columns (Table 6). Replace all $f_{l}$ in the formula cited in Table 6 with $f_{l,a}$ the above in this paper and consider the influence of PVC constraints and CFRP strip spacing. The comparison between the predicted results of different models and experimental data is shown in Fig. 13. The models have large deviations in estimating the ultimate compressive stress and ultimate strain of CFRP–PVC columns. The determination of the ultimate stress $f_{cc}$ and ultimate strain $\varepsilon_{cc}$ was essential for the stress–strain of composite column predictions. Therefore, the ultimate stress and strain needed to be corrected.

14 $$\frac{{f_{cc} }}{{f_{co} }} = 1 + 3.3\left( {\lambda \frac{{f_{l} }}{{f_{co} }}} \right)^{\alpha } \;{\text{and}}$$

15 $$\frac{{\varepsilon_{cu} }}{{\varepsilon_{co} }} = 1.75 + k\left( {\frac{{f_{l,a} }}{{f_{co} }}} \right)\left( {\frac{{\varepsilon_{frp} }}{{\varepsilon_{co} }}} \right)^{\beta } .$$

**Table 6:** Ultimate strength and strain models of FRP confined concrete.

Models	Ultimate strength expressions	Ultimate strain expressions
Lam and Teng^44^	$\frac{{f_{cc} }}{{f_{co} }} = 1 + 3.3\frac{{f_{l,a} }}{{f_{co} }}$	$\frac{{\varepsilon_{cu} }}{{\varepsilon_{co} }} = 1.75 + 5.53\left( {\frac{{f_{l,a} }}{{f_{co} }}} \right)\left( {\frac{{\varepsilon_{frp} }}{{\varepsilon_{co} }}} \right)^{0.45}$
Wei and Wu^45^	$\frac{{f_{cc} }}{{f_{co} }} = 1 + 2.2\left( {\frac{{f_{l} }}{{f_{co} }}} \right)^{0.94}$	$\frac{{\varepsilon_{cu} }}{{\varepsilon_{co} }} = 1.75 + 12\left( {\frac{{f_{l} }}{{f_{co} }}} \right)^{0.75} \left( {\frac{{f_{30} }}{{f_{co} }}} \right)^{0.62}$
Youssef et al.^46^	$\frac{{f_{cc} }}{{f_{co} }} = 1 + 2.25\left( {\frac{{f_{l} }}{{f_{co} }}} \right)^{\frac{5}{4}}$	$\varepsilon_{cu} = 0.003368 + 0.259\left( {\frac{{f_{l} }}{{f_{co} }}} \right)\left( {\frac{{f_{frp} }}{{E_{frp} }}} \right)^{\frac{1}{2}}$

**Figure 14:**
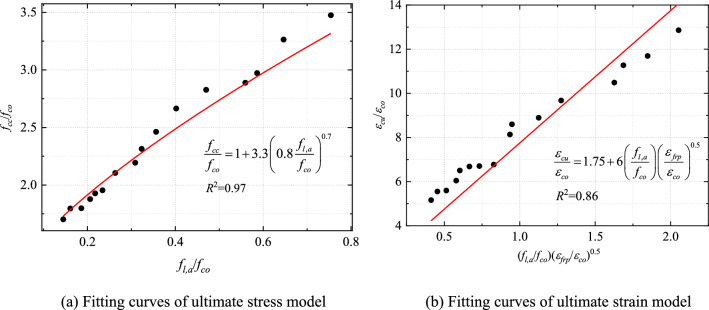
Final model fitting curve.

Based on Lam and Teng's ultimate stress and strain model^44^, as shown in Eqs. (14) and (15), regression analysis and iterative calculations of the test results were performed to determine the fitting coefficients $\lambda$ = 0.8 $\alpha$ = 0.7, $k$ = 6 $\beta$ = 0.5, and *R*^2^ = 0.97 *R*^2^ = 0.86 (Fig. 14). The strength and ultimate strain models of CFRP–PVC–SAC were expressed by Eqs. (16) and (17) as

16 $$\frac{{f_{cc} }}{{f_{co} }} = 1 + 3.3\left( {0.8\frac{{f_{l} }}{{f_{co} }}} \right)^{0.7} \;{\text{and}}$$

17 $$\frac{{\varepsilon_{cu} }}{{\varepsilon_{co} }} = 1.75 + 6\left( {\frac{{f_{l,a} }}{{f_{co} }}} \right)\left( {\frac{{\varepsilon_{frp} }}{{\varepsilon_{co} }}} \right)^{0.5} .$$

**Figure 15:**
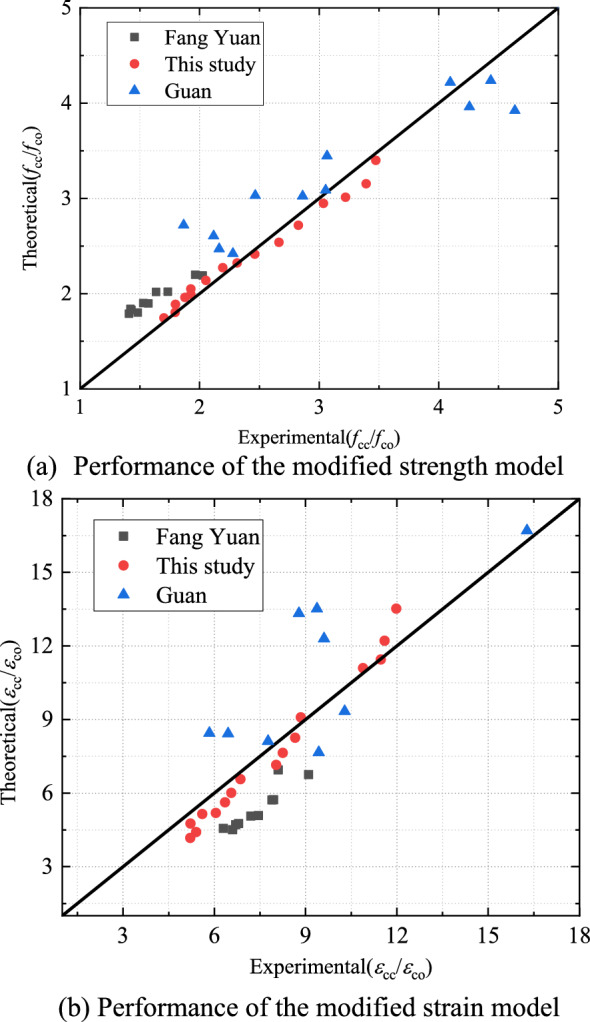
Modified model performance.

Based on the above ultimate stress and strain equations, a comparison of the experimental and predicted ultimate stresses and ultimate axial strains was made (Fig. 15). Quoting the experimental data of Fang^39^ and Guan^13^, the predicted values were seen to be based on the ultimate stress–strain model developed here and in good agreement with the experimental ultimate stress–strain values with less deviation.

### Stress–strain model for CFRP-constrained SAC

In this section, based on the stress–strain model of Lam and Teng^44^, the ultimate stress $f_{cc}$ and ultimate strain $\varepsilon_{cc}$ were modified by considering the effects of CFRP strip spacing and aggregate properties, and the stress–strain model of the CFRP–PVC–SAC column was established, as shown in the following equation.

18 $$\sigma_{c} = E_{c} \varepsilon_{c} - \frac{{\left( {E_{c} - E_{2} } \right)^{2} }}{{4f_{o} }}\varepsilon_{c}^{2} \quad {\text{for}}\;0 \le \varepsilon_{c} \le \varepsilon_{t} ,$$

19 $$\sigma_{c} = f_{o} + E_{2} \varepsilon_{c} \quad {\text{for}}\;\varepsilon_{t} \le \varepsilon_{c} \le \varepsilon_{cu} ,$$

20 $$\varepsilon_{t} = \frac{{2f_{o} }}{{(E_{c} - E_{2} )}},$$

21 $$E_{2} = \frac{{f_{cc} - f_{o} }}{{\varepsilon_{cu} }},$$

22 $$\varepsilon_{cc} = \frac{{f_{cc} - f_{o} }}{{E_{2} }},\;{\text{and}}$$

23 $$f_{o} = f_{co} ,$$

where $\sigma_{c}$ and $\varepsilon_{c}$ denote the axial stress and strain in the confined concrete, $f_{o}$ the intercept of the stress axis by the linear second part, $E_{2}$ the slope of the linear second part, $f_{cc}$ the compressive strength of the compressive concrete, and $\varepsilon_{cu}$ the ultimate strain.

**Figure 16:**
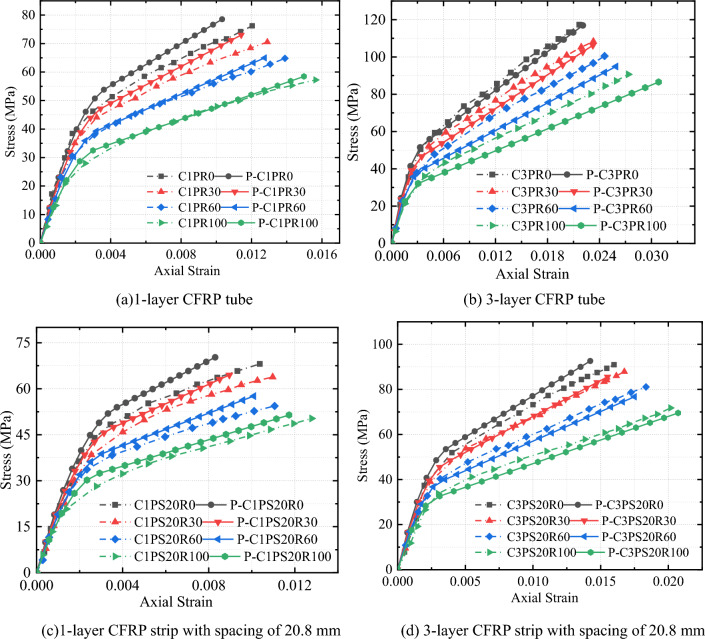
Comparison of predicted stress–strain curves with test data.

The existing FRP-constrained normal concrete model could not be directly applied to FRP-constrained SAC. As mentioned before, the porosity, low density, high water absorption, and fragility of SCG led to different mechanical properties of SAC under CFRP–PVC confinement. Based on the Lam and Teng stress–strain model, the axial stress–strain model of CFRP–PVC–SAC was developed. The stress–strain prediction curves of the CFRP–PVC–SAC model showed that the model agreed well with the experimental data (Fig. 16).

## Conclusions

Tests investigated the stresses of 8 unconfined short columns and 32 CFRP–PVC–SAC short columns under axial pressure and the effects of CFRP strip spacing and SCG replacement proportion considered. The following conclusions were drawn.

1. Under the constraint of CFRP–PVC, the strength of coal gangue concrete increased by 1.68 to 3.48 times, and the ultimate strain increased by 5.21–11.98 times. Under the same constraint form, the bearing capacity of the specimen decreases with an increase in the replacement rate of coal gangue, and the ultimate strain is positively correlated with the replacement rate.
2. With an increase in CFRP effective constraint, the bearing capacity and ultimate compressive strain of the specimen also increase. Compared to CFRP strips with a spacing of 20 mm, the CFRP full-wrapped form increases the strength of the specimen by 10.5–29.0%, and the axial strain increases by 16.1–39.0%. Although the constraint stiffness of the CFRP strip–PVC constraint form is low, it still has a strong constraint effect on the core concrete.
3. The stress–strain curves of CFRP–PVC–SAC short columns can be roughly divided into two stages. In general, the initial stage is similar to unconstrained concrete. In the second stage of the stress–strain relationship, the slope of the stress–strain curve gradually increases with the increase in the effective constraint of CFRP.
4. Based on a large amount of the latest experimental data, including the present measured data and literature, a modified model was developed to facilitate the prediction of the stress–strain relationship for CFRP–PVC–SAC short columns. The model's prediction results were in good agreement with the experimental data.

Although the modifications to the model thoroughly consider the impact of the FRP confinement form and aggregate characteristics on the mechanical properties of the test specimens, it should be noted that the model is generated based on a limited database. Therefore, it is strongly recommended to conduct further research to include various types of FRP-confined SAC specimens to obtain a more accurate model. Simultaneously, it is necessary to address the interface issues between FRP and PVC and delve deeper into the investigation of the spacing between FRP strips.

## Data Availability

All data supporting the findings in this study are available from the corresponding author upon reasonable request.

## References

[1] Li J, Wang J (2019). Comprehensive utilization and environmental risks of coal gangue: A review. J. Clean. Prod..

[2] Stracher GB, Taylor TP (2003). Coal fires burning out of control around the world: Thermodynamic recipe for environmental catastrophe. Int. J. Coal. Geol..

[3] Jabłońska B, Kityk AV, Busch M, Huber P (2017). The structural and surface properties of natural and modified coal gangue. J. Environ. Manag..

[4] Tang Q, Li L, Zhang S, Zheng L, Miao C (2018). Characterization of heavy metals in coal gangue-reclaimed soils from a coal mining area. J. Geochem. Explor..

[5] Dong Z, Xia J, Fan C, Cao J (2015). Activity of calcined coal gangue fine aggregate and its effect on the mechanical behavior of cement mortar. Constr. Build. Mater..

[6] Koshy N, Dondrob K, Hu L, Wen Q, Meegoda JN (2019). Synthesis and characterization of geopolymers derived from coal gangue, fly ash and red mud. Constr. Build..

[7] Zhang Y, Wang Q, Zhou M, Fang Y, Zhang Z (2020). Mechanical properties of concrete with coarse spontaneous combustion gangue aggregate (SCGA): Experimental investigation and prediction methodology. Constr. Build. Mater..

[8] Guo Y, Zhao Q, Yan K, Cheng F, Lou HH (2014). Novel process for alumina extraction via the coupling treatment of coal gangue and bauxite red mud. Ind. Eng. Chem. Res..

[9] Zhang Y, Ling T-C (2020). Reactivity activation of waste coal gangue and its impact on the properties of cement-based materials—A review. Constr. Build. Mater..

[10] Cong XY, Lu S, Yao Y, Wang Z (2016). Fabrication and characterization of self-ignition coal gangue autoclaved aerated concrete. Mater. Des..

[11] Li Y, Liu S, Guan X (2021). Multitechnique investigation of concrete with coal gangue. Constr. Build. Mater..

[12] Gao S, Guo L (2020). Utilization of coal gangue as coarse aggregates in structural concrete. Constr. Build. Mater..

[13] Guan H, Xia Y, Zhang S, Wang J (2022). Stress–strain behaviour of spontaneous combustion gangue coarse aggregate concrete under FRP tube confinement. Constr. Build. Mater..

[14] Zhao H, Ren T, Remennikov A (2021). Behaviour of FRP-confined coal reject concrete columns under axial compression. Compos. Struct..

[15] Zhou M, Dou Y, Zhang Y, Zhang Y, Zhang B (2019). Effects of the variety and content of coal gangue coarse aggregate on the mechanical properties of concrete. Constr. Build. Mater..

[16] Wang Q, Li Z, Zhang Y, Zhang H, Zhou M, Fang Y (2020). Influence of coarse coal gangue aggregates on elastic modulus and drying shrinkage behaviour of concrete. J. Build. Eng..

[17] Wang C, Ni W, Zhang S, Wang S, Gai G, Wang W (2016). Preparation and properties of autoclaved aerated concrete using coal gangue and iron ore tailings. Constr. Build. Mater..

[18] Wang W, Sheikh MN, Al-Baali AQ, Hadi MNS (2018). Compressive behaviour of partially FRP confined concrete: Experimental observations and assessment of the stress–strain models. Constr. Build. Mater..

[19] Jiang S-F, Ma S-L, Wu Z-Q (2014). Experimental study and theoretical analysis on slender concrete-filled CFRP–PVC tubular columns. Constr. Build. Mater..

[20] Yu F, Xu G, Niu D, Cheng A, Wu P, Kong Z (2018). Experimental study on PVC-CFRP confined concrete columns under low cyclic loading. Constr. Build. Mater..

[21] Ozbakkaloglu T, Lim JC (2013). Axial compressive behavior of FRP-confined concrete: Experimental test database and a new design-oriented model. Compos. Part. B Eng..

[22] Zeng JJ, Guo Y-C, Gao W-Y, Li J-Z, Xie J-H (2017). Behavior of partially and fully FRP-confined circularized square columns under axial compression. Constr. Build. Mater..

[23] Zhao JL, Yu T, Teng JG (2014). Stress–strain behavior of FRP-confined recycled aggregate concrete. J. Compos. Constr..

[24] Zhou Y, Liu X, Xing F, Cui H, Sui L (2016). Axial compressive behavior of FRP-confined lightweight aggregate concrete: An experimental study and stress-strain relation model. Constr. Build. Mater..

[25] Zeng J-J, Zhang X-W, Chen G-M, Wang X-M, Jiang T (2020). FRP-confined recycled glass aggregate concrete: Concept and axial compressive behavior. J. Build. Eng..

[26] Gupta PK, Verma VK (2016). Study of concrete-filled unplasticized poly-vinyl chloride tubes in marine environment. Proc. Inst. Mech. Eng. M J. Eng..

[27] Fakharifar M, Chen G (2017). FRP-confined concrete filled PVC tubes: A new design concept for ductile column construction in seismic regions. Constr. Build. Mater..

[28] Zhang H, Hadi MNS (2019). Geogrid-confined pervious geopolymer concrete piles with FRP-PVC-confined concrete core: Concept and behaviour. Constr. Build. Mater..

[29] Alves LM, Martins P (2009). Cold expansion and reduction of thin-walled PVC tubes using a die. J. Mater. Process. Technol..

[30] Feng C, Yu F, Fang Y (2021). Mechanical behavior of PVC tube confined concrete and PVC-FRP confined concrete: A review. Structures.

[31] Fakharifar M, Chen G (2016). Compressive behavior of FRP-confined concrete-filled PVC tubularcolumns. Compos. Struct..

[32] Abdulla NA (2017). Concrete filled PVC tube: A review. Constr. Build. Mater..

[33] Wang J-Y, Yang Q-B (2012). Investigation on compressive behaviors of thermoplastic pipe confined concrete. Constr. Build. Mater..

[34] Askari SM, Khaloo A, Borhani MH, Masoule MST (2020). Performance of polypropylene fiber reinforced concrete-filled UPVC tube columns under axial compression. Constr. Build. Mater..

[35] Toutanji H, Saafi M (2001). Durability studies on concrete columns encased in PVC–FRP composite tubes. Compos. Struct..

[36] Bandyopadhyay, Maurya KK, Samanta AK (2020). Investigation on UPVC confined RC columns with recycled aggregate concrete using C&D waste. Structures.

[37] Yu F, Li D, Niu D, Zhu D, Kong Z, Zhang N, Fang Y (2019). A model for ultimate bearing capacity of PVC-CFRP confined concrete column with reinforced concrete beam joint under axial compression. Constr. Build. Mater..

[38] Gao C, Huang L, Yan L, Jin R, Kasal B (2019). Strength and ductility improvement of recycled aggregate concrete by polyester FRP-PVC tube confinement. Compos. Part B Eng..

[39] Fang Y, Yu F, Guan Y, Wang Z, Feng C, Lia D (2020). A model for predicting the stress–strain relation of PVC-CFRP confined concrete stub columns under axial compression. Structures.

[40] People’s Republic of China Industry Standard (2006). Standard for Technical Requirements and Test Method of Sand and Crushed Stone (or Gravel) for Ordinary Concrete (JGJ52-2006).

[41] People’s Republic of China Industry Standard (2011). Specification for Mix Proportion Design of Ordinary Concrete (JGJ 55-2011).

[42] ASTM D. *Standard Test Method for Tensile Properties of Polymer Matrix Composite Materials* (2008).

[43] Yu F, Niu D (2013). Experimental study on PVC-CFRP confined reinforced concrete short column under axial compression. J. Build. Struct..

[44] Lam L. & Teng J.G. Design-oriented stress–strain model for FRP-confined concrete. *Constr Build Mater.* **17**(6-7), 471–489. 10.1016/S0950-0618(03)00045-X (2003).

[45] Wei, Y.-Y. & Wu, Y.-F. Unified stress–strain model of concrete for FRP-confined columns. *Constr Build Mater.* **26**(1), 381–392. 10.1016/j.conbuildmat.2011.06.037 (2012).

[46] Youssef, M. N., Feng, M. Q. & Mosallam,A. S. Stress–strain model for concrete confined by FRP composites. *Composites Part B: Engineering* **38**(5-6), 614–628. 10.1016/j.compositesb.2006.07.020 (2007).

